# Genomic surveillance and evolution of co-circulating avian influenza H5N1 and H5N8 viruses in Egypt, 2022–2024

**DOI:** 10.1080/22221751.2025.2562046

**Published:** 2025-09-15

**Authors:** Samah Eid, Naglaa M. Hagag, Zienab Mosaad, Neveen R. Bakry, Mohamed H. Elhusseiny, Wesam H. Mady, Ahmed Erfan, Fatma Amer, Osama Mahana, Nahed Yehia, Moataz M. Elsayed, Dalia Said, Afaf Abdelbaset, Ahlam E. Yonis, Reem M. Reda, Mohamed A. Saif, Ahmed I. Abdelmawgoud, Tamer A. El-Aried, Mahmoud Said, Abdullah Selim, Eman Farghaly, Abdelsatar Arafa, Momtaz Shahein, Mahmoud M. Naguib

**Affiliations:** a Reference Laboratory for Veterinary Quality Control on Poultry Production, Animal Health Research Institute, Agriculture Research Center, Giza, Egypt; b Department of Medical Biochemistry and Microbiology, Zoonosis Science Center, Uppsala University, Uppsala, Sweden; c Department of Infection Biology and Microbiomes, Institute of Infection, Veterinary and Ecological Sciences, University of Liverpool, Liverpool, UK

**Keywords:** Avian influenza, H5, virus evolution, genetic diversity, Egypt, poultry

## Abstract

For over two decades, avian influenza virus (AIV) has significantly impacted the Egyptian poultry population, with multiple subtypes and genotypes contributing to significant economic and agricultural losses. As part of an ongoing national surveillance effort, this study aimed to monitor and genetically characterize AIV circulation across various poultry sectors in Egypt. Between 2022 and 2024, a total of 446,790 swab samples were collected, representing commercial farms (*n* = 25,057), backyard flocks (*n* = 403), and live bird markets “LBM” (*n* = 1250) to assess the prevalence and genetic diversity of circulating AIV strains. A total of 173 sampling units were found positive for high pathogenicity (HP) AIV H5, including farms (*n* = 17), backyards (*n* = 11), and LBMs (*n* = 145). The HPAIV of H5N8 subtype was dominant (*n* = 75) over the H5N1 (*n* = 27) subtypes among all sectors and bird species (chickens, ducks, turkeys). Whole genome sequence analysis of positive H5 samples revealed high similarity with HPAIVs of clade 2.3.4.4b, which has been confirmed phylogenetically. Two distinct subtypes H5N1 (EA-2021-AB genotype) and H5N8 (EA-2020-A genotype) were identified, with two variants detected within the H5N8 viruses. Evolutionary analyses indicate that Egyptian H5N8 viruses are under strong selection pressure and exhibit a higher nucleotide substitution rate compared to the Egyptian H5N1 viruses of clade 2.3.4.4b. With the evolving HPAI H5 virus’s situation in different locations around the globe, including Egypt, this study underlines the importance of active surveillance in the timely detection of emerging AIV genotypes, monitoring virus evolution, and refining risk assessments.

## Introduction

Avian influenza virus (AIV), particularly H5 subtype, continues to evolve through point mutations or genomic segment reassortment, resulting in the emergence of novel variants that pose a significant risk to both animal and human health. High pathogenicity AIV (HPAIV) H5N8 of clade 2.3.4.4 was first detected in China in 2010 in China as a result of genetic reassortment of different AIV subtypes [1]. Further in early 2014, a novel reassortant of HPAIV H5N8, classified as clade 2.3.4.4a, was reported in South Korea [2] and subsequently spread via migratory birds to Europe and North America, causing multiple outbreaks [3–5]. During the 2016/2017 season, new reassortants of HPAIV H5N8 virus with distinct gene constellations, belonging to clade 2.3.4.4b, were identified in wild birds across several countries [6–8]. Since 2021, the HPAIV H5Nx of clade 2.3.4.4b, particularly H5N1, has rapidly expanded, affecting numerous countries with different genotypes and causing an unprecedented number of outbreaks across diverse avian and mammalian species [9]. During the current panzootic, hundreds of outbreaks in wild birds have been resulting in millions of deaths, and almost 50 mammalian species have tested positive for HPAIV H5N1 [10].

In Egypt, HPAIV H5N1 was first detected in December 2005 and was phylogenetically classified as clade 2.2.1. Viruses of this clade continued to spread until 2017, when they were replaced by HPAIV H5N8, which had emerged in late 2016 [11]. Phylogenetic analysis of the Egyptian H5N8 revealed a close relationship to clade 2.3.4.4b strains identified in Russia during the same year [12]. Within a short period, the virus spread extensively among domestic poultry across different governorates, posing a significant threat to the Egyptian poultry industry [13,14]. Additionally, reassortment events between the Egyptian HPAIV H5N8 and the low pathogenicity avian influenza virus (LPAIV) H9N2 subtype led to the emergence of novel HPAI H5N2 viruses in 2018/2019 [15,16]. Later in 2021, HPAIV H5N1 of clade 2.3.4.4b was found in both domestic and wild birds in Egypt [17,18]. Most recently, HPAV H5N1 was reported in wild rats in rural areas in Egypt close to poultry farms and markets [19]. The recurrence of HPAIV H5 outbreaks in birds in Egypt has raised concern about the potential spillover to livestock and other wild animals. These findings highlight the ongoing genetic evolution and global spread of HPAI H5 viruses, underscoring the need for continuous monitoring and control efforts.

In this study, we conducted a comprehensive genomic and epidemiological investigation of AIV H5 in Egypt, analysing approximately half a million swab samples collected from various bird sectors (farms, backyards, and live bird markets) and bird species (chickens, ducks, geese, and turkeys) between 2022 and 2024. Genetic characterization and evolutionary analysis were performed on positive samples to assess viral diversity and transmission dynamics.

## Materials and methods

### Sampling and ethical consideration

**Figure 1.**
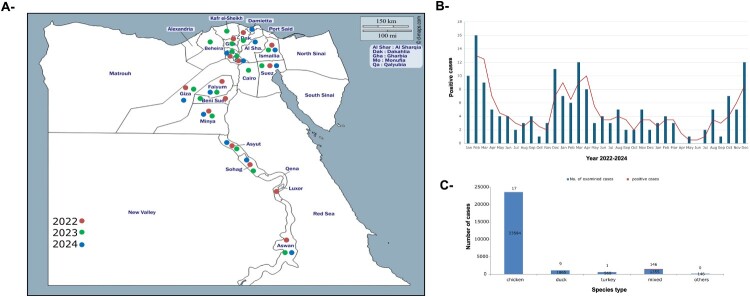
Surveillance and epidemiological features of A(H5) viruses in Egypt, 2022-2024. (A) Geographical distribution of A(H5) viruses in Egypt during 2022–2024 (B) Distribution of positive H5 per month over 2022-2024. (C) Number of samples collected from each species indicating total tested and positively detected for H5 virus. “Others” indicates other sources rather than chicken, duck, and turkey (e.g. environmental samples).

A total of 396,173 oropharyngeal and cloacal swab samples were obtained from 25,057 poultry farms across Egypt, including chickens, ducks, and turkeys. Farms were selected in collaboration with governmental veterinary authorities in Egypt to ensure sectoral representation, covering all Egyptian governorates (Supplementary table 1) and farms of different sizes (small, medium, and large). Sampling was conducted by trained and authorized veterinarians independent of farm owners. Birds were randomly selected within flocks to minimize bias. The number of swabs per farm ranged from five to 10 swabs, depending on birds’ number per farm. Additionally, 3768 swabs were obtained from 403 backyard farms, and 46,849 swabs were collected from 1250 live bird markets (LBMs). Five to 20 individual tracheal and/or cloacal swabs from each sector were combined as one sample. Samples were collected from all 27 Egyptian governorates across both Upper and Lower Egypt (Figure 1, Supplementary Table 1). These samples were collected as part of an active AIV surveillance program in Egypt conducted by the Reference Laboratory of Veterinary Quality Control on Poultry Production (RLQP), Animal Health Research Institute, Egypt, and the General Organization for Veterinary Services (GOVs) [20], under approval number 20220101. Epidemiological information for all collected samples in the supplementary table S1.

### Virus detection and isolation

Viral RNA was extracted from each pooled specimen using the QIAamp Viral RNA Mini Kit (Qiagen, Hilden, Germany) following the manufacturer’s instructions. All RNA extracts were initially screened for the influenza A virus matrix (M) gene using a real-time reverse transcription polymerase chain reaction (RT-qPCR) assay [21]. Samples testing positive for influenza M gene were subsequently analysed using subtype-specific RT-qPCR assays for the hemagglutinin (HA) and neuraminidase (NA) genes of the AIV H5, H9, and N1, N2, N8, respectively [22]. RT-qPCR reactions were run on the MX3005P system (Agilent, Santa Clara, CA, USA).

For virus isolation, original pooled samples that tested RT-qPCR positive for influenza were inoculated into the allantoic cavity of 10–12 d-old specific-pathogen-free (SPF) embryonated chicken eggs (ECEs) following standard protocols of the World Organisation for Animal Health [23]. Allantoic fluid was collected from inoculated eggs 36–48 hours post inoculation. The presence of AIV was confirmed by hemagglutination assay with 1% chicken red blood cells and subsequently verified by RT-qPCR for the AIV HA and NA genes.

### Genetic and phylogenetic characterization

The HA and NA gene segments were successfully amplified from 38 and 13 positive isolates, respectively, using sets of primers as outlined by Hoper et al. (2011) [24]. Additionally, full genome amplification was conducted for five representative H5 viruses. Samples for whole genome sequencing were selected to cover different sectors (farms, backyards, and LBM), as well as representing recently circulating strains from 2024. Briefly, following RT–PCR amplification, the target products were separated by agarose gel electrophoresis, excised, and purified from gels using the QIAquick Gel Extraction Kit (Qiagen, Hilden, Germany). The purified PCR products were then used for sequencing reactions with the BigDye Terminator v3.1 Cycle Sequencing Kit (Applied Biosystems, Waltham, MA, USA). The sequencing reactions were purified using the Centrisep spin column (Thermo Fisher, Waltham, MA, USA) and processed 3500 XL Genetic Analyzer (Life Technologies, Carlsbad, CA, USA). The resulting sequences were assembled and edited using Geneious Prime 2025.1.2 (https://www.geneious.com). A Blast search was performed using the Basic Local Alignment Search Tool (BLASTn) at NCBI https://blast.ncbi.nlm.nih.gov/Blast.cgi. Sequences generated in this study were deposited at the GISAID (the Global Initiative on Sharing All Influenza Data) platform under accession numbers listed in supplementary table 2. Two of those five samples were sequenced using Illumina sequencing platform NovaSeq 6000 after library preparation, as previously described at [25]

Identifying the genotype of each strain of H5N1 or H5N8 was performed using the Genin2 software https://github.com/izsvenezie-virology/genin2. The analysis was performed using sequences generated in this study (H5N1 = 3 and H5N8 = 2) and publicity available sequences (H5N1 = 14 and H5N8 = 101). To conduct a comparative phylogenetic analysis, reference sequences of HPAIV H5 viruses from clades 2.3.4.4b, along with Egyptian HPAIV H5N1/H5N8 strains, were downloaded from the GISAID database (GISAID, http://www.gisaid.org, accessed 01 May 2025). These sequences were selected based on geographic distribution and availability of complete genome data to ensure representative coverage. The retrieved sequences, along with those from the current study, were aligned using MAFFT (Multiple Alignment using Fast Fourier Transform) [26]. Phylogenetic relationships for all gene segments were inferred using the maximum likelihood method, with the best-fit model selected based on the Akaike Information Criterion (AIC) in IQ-TREE software version 1.1.3 [27]. The resulting phylogenetic trees were annotated and visualized using FigTree v1.4.2 software (http://tree.bio.ed.ac.uk/software/figtree/, accessed on 01 May 2025) and Inkscape 1.4.2 (https://inkscape.org).

### Bayesian evolutionary inference and selection pressure

Bayesian Evolutionary Analysis Sampling Trees (BEAST v1.10.4) was used to estimate changes in effective population size of the H5N1 (clade 2.3.4.4b) and H5N8 (clade 2.3.4.4b) viruses since their introduction into Egypt. We applied a generalized time-reversible (GTR) model + I (invariant sites) for nucleotide substitution, incorporating gamma-distributed site rate variation. An uncorrelated relaxed molecular clock with a lognormal distribution was used. To ensure robust parameter estimation, we performed three separate Markov chain Monte Carlo (MCMC) simulations, each consisting of 50 million steps, with samples collected every 5,000 iterations for the HA of H5N1 (*n* = 35) and HA of H5N8 (*n* = 213) gene segments. Independent runs were combined using LogCombiner, and parameter convergences were assessed in Tracer v.1.7.1, ensuring ESS (Effective Sample Size) > 200. Mean substitution rates and 95% highest posterior density (HPD) intervals were recorded.

To assess selection pressure on the Egyptian H5 viruses, codon-based analyses were performed on multiple sequence alignments of HPAI H5N1 and H5N8 viruses using the Datamonkey 2.0 platform [28]. The dataset comprised all HA sequences available and obtained from poultry for H5N1 (*n* = 35) and H5N8 (*n* = 213) with a minimum length of 1600 bp. Sites under positive selection were identified by a dN/dS ratio greater than 1.0 with a significance threshold of *p* < 0.1. Both pervasive and episodic selection were examined using the Fixed Effects Likelihood (FEL) and Mixed Effects Model of Evolution (MEME) methods, respectively.

## Results

### Active surveillance

A total of 370 samples were found positive for the influenza A virus M gene. Of these, 173 samples were confirmed as HPAI H5 viruses, including 75 H5N8, 27 H5N1, and 71 H5Nx viruses (H5-positive but not fully subtyped due to low virus RNA concentration and failure to isolate the virus). The remaining 197 samples were identified as LPAI H9 viruses. Only 17 commercial farms were tested positive for the influenza H5 subtype (chickens = 11, ducks = 5, turkeys = 1) out of 25,057 apparently healthy birds examined farms as part of an active surveillance program in Egypt between 2022 and 2024. These influenza positive farms were distributed in 10 out of 27 governorates, in North and South of Egypt during 2022–2024, representing a geographical prevalence 37.03% (Supplementary table 1). In addition, 11 and 145 samples tested positive for HPAI H5Nx viruses in backyards and LBMs, respectively, out of 403 and 1250 sectors sampled (Figure 1). A clear seasonal variation was observed over this three-year period where high numbers of positive cases were detected during the winter season, from late November to April (Figure 1). The 197 H9-positive samples were orginated from commercial farms (*n* = 133), LBMs (*n* = 61), and backyard flocks (*n* = 3).

### Notable features of the Egyptian H5 viruses

The hemagglutinin (HA) proteins of Egyptian H5 viruses analysed in this study exhibited a multibasic cleavage site motif, PLREKRRKR/GLF, consistent with a highly pathogenic avian influenza (HPAI) phenotype. Analysis of the receptor-binding pocket revealed conserved residues H107, N193, G225, Q226, and G228 (H3 numbering), indicative of a binding preference for avian-type α2,3-linked sialic acid receptors [29,30] (Table 1 and supplementary table 3). Several mutations previously associated with enhanced binding affinity to human-type receptors *in vitro* were detected. Specifically, S137A, S158N, and T160A were present in the vast majority of Egyptian H5Nx viruses circulating between 2022 and 2024. Additionally, mutations such as P140S, A188 V, and K216R were observed only sporadically in the HA of the Egyptian H5Nx viruses. Notably, Egyptian HPAI H5N1 viruses harboured K189 and R372 in their HA sequences, distinguishing them from H5N1-A/duck/Saratov/29-02 V/2021 and local HPAI H5N8 viruses. Furthermore, the HA sequences of Egyptian HPAI H5N8 viruses revealed at least two distinct variants, differentiating strains from 2017 to 2022 and 2019 to 2024, based on amino acid changes at positions 144A/T, 188V/A, 240N/D, 272E/G, and 507G/S. Further, genome analysis of the Egyptian HPAI H5N8 viruses sequenced in this study revealed the PA-K356R mutation, which has been previously described to enhance adaptation to mammalian hosts (Table 1) [31]. No E627K and D701N substitutional mutations were observed in the PB2 of any of the H5N1 and H5N8 sequenced viruses (Table 1) [31]. Additionally, no substitutional amino acid mutations related to Baloxavir (I38V/M and E199G), oseltamivir (H274Y, R292K, and N294S – N2 numbering), or amantadine (L26F/I, V27A/T/I, A30T/V, S31N, and G34E/R) resistance were detected in the PA, NA and MMP respectively (Table 1) [32–35].

**Table 1.** Key amino acid analysis among different proteins of the Egyptian HPAI H5 viruses sequenced in this study against reference strains.

	HA (H3 numbering)								Oseltamivir RM^a^	PB2		PA	Amantadine RM	NS1 length
	Receptor binding sites							Cleavage site						
	107	133	193	216	225	226	228			627	701	356		
A/duck/Chelyabinsk/1207-1/2020^b^	H	L	N	K	G	Q	G	PLREKRRKRGLF	No	E	D	K	No	230
A/duck/Egypt/F76/2024	H	L	N	**R**^c^	G	Q	G	PLREKRRKRGLF	No	E	D	**R**	No	230
A/chicken/Egypt/F78/2024	H	L	N	**R**	G	Q	G	PLREKRRKRGLF	No	E	D	**R**	No	230
A/duck/Saratov/29-02 V/2021^d^	H	L	N	K	G	Q	G	PLREKRRKRGLF	No	E	D	K	No	230
A/geese/Egypt/FAO-S155/2024	H	L	**K**	K	G	Q	G	PLREKRRKRGLF	No	E	D	K	No	230
A/geese/Egypt/S8/2024	H	L	**K**	K	G	Q	G	PLREKRRKRGLF	No	E	D	K	No	230
A/chicken/Egypt/S42/2024	H	L	**K**	K	G	Q	G	PLREKRRKRGLF	No	E	D	K	No	230

^a^ RM = resistance markers.

^b^ Reference strain of genotype EA-2020-A (H5N8).

^c^ Bold means different compared to the reference strain.

^d^ Reference strain of genotype EA-2021-AB (H5N1).

### Different subtypes and genotypes circulating in Egypt

Genotype identification was performed using the Genin2 software https://github.com/izsvenezie-virology/genin2. The Egyptian H5N1 viruses in this study were classified as “EA-2021-AB” H5N1-A/duck/Saratov/29-02 V/2021-like, whereas the Egyptian H5N8 viruses were identified as “EA-2020-A” H5N8-A/duck/Chelyabinsk/1207-1/2020-like (Supplementary table 4). Further analysis, including publicity available H5 whole genome sequence of clade 2.3.4.4b from Egypt, revealed the presence of several genotypes in Egypt including EA-2020-A (H5N8), EA-2021-Q (H5N8), EA-2021-AB(H5N1), and EA-2020-C (H5N1) (Supplementary tables 5 and 6).

**Figure 2.**
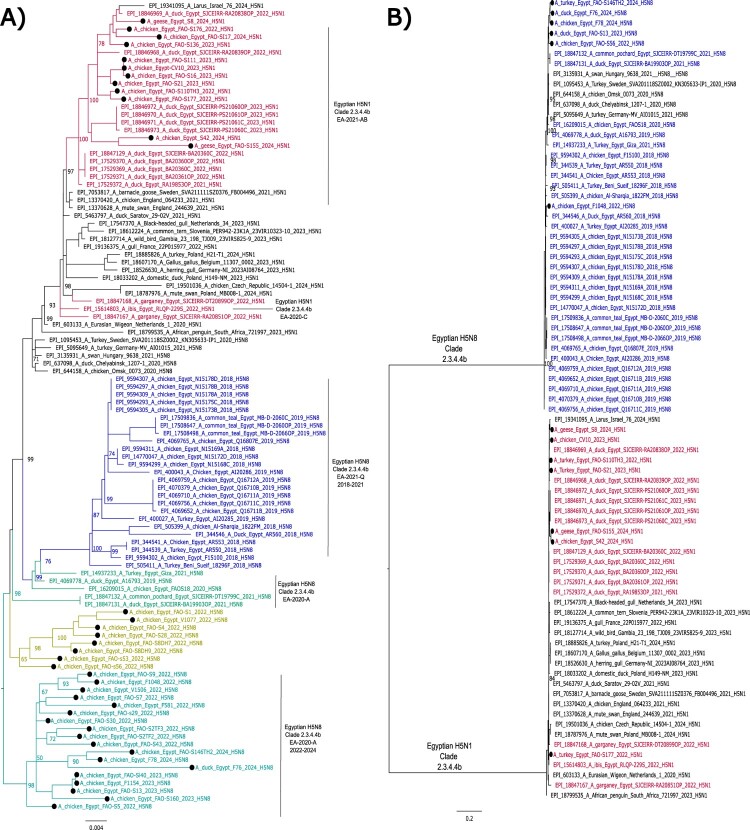
Phylogenetic tree of the HA (A) and NA (B) gene segments of HPAI H5N1 (red) and H5N8 viruses from Egypt (blue, green, cyan and brown). Viruses generated in this study is shown by black dots. Representative reference strains from different genotypes are shown in black colour. Trees were generated, after the selection of the best-fitted model, by employing maximum likelihood methodology based on Akaike criterion using IQ-tree software version 1.1.3 [27].

Phylogenetic analyses were consistent with the genetic characterization and showed that the Egyptian HPAI H5N8 viruses clustered with EA-2020-A″ H5N8-A/duck/Chelyabinsk/1207-1/2020-like. While the HA of the Egyptian HPAI H5N1 viruses are grouped with EA-2021-AB″ H5N1-A/duck/Saratov/29-02 V/2021-like viruses (Figure 2). Phylogenies of the remaining genes revealed close relatedness of the Egyptian to the recent European HPAI H5N8 viruses from 2022 to 2024 of genotypes EA-2020-A (H5N8) and EA-2021-AB (H5N1). (Supplementary figure 1).

### The H5N8 subtype reveals higher evolutionary rates and selection pressure

The estimated evolutionary rate, based on combined three independent runs, for the HA gene of the Egyptian H5N8 viruses of clade 2.3.4.4b was 5.87 × 10^−3^ substitutions per site/year [95% highest posterior density (HPD), 4.89 × 10^−3^–6.83 × 10^−3^] while the estimated evolutionary rate for the Egyptian HPAI H5N1 viruses of clade 2.3.4.4b was found 4.87 × 10^−3^ (95% HPD, 3.14 × 10^−3^–6.73 × 10^−3^).

Further, implementing MEME method to detect episodic diversifying selection, positive selection pressure for hemagglutinin was found at one amino acid residue of the H5N1 and 9 sites at the H5N8 (Supplementary Table 7). In addition, FEL analysis, to estimate site-specific selection, revealed two AA sites at the HA of H5N8 viruses and no sites at the HA of the H5N1 viruses with diversifying selection dN > dS. Those two AA sites (137 and 163 – H3 numbering) were detected by both methods in the HA of the H5N8, meaning that amino acid changes at these sites may confer an advantage and are undergoing adaptive evolution.

## Discussion

The ongoing spread of HPAI viruses, particularly H5 subtypes, presents a serious threat to animal and public health around the globe [9,36,37]. In Egypt, different subtypes and genotypes of HPAI H5 viruses have been isolated from birds, particularly H5N1 [18], H5N2 [15,38], and H5N8 [20], in addition to a recent report of HPAI H5N1 virus in wild rats [19]. In this study, we investigated the molecular epidemiology and explored the genetic evolution of HPAI H5 of H5N1 and H5N8 subtypes co-circulating in Egypt between 2022 and 2024. The findings of this study highlight the complexity of the HPAI H5 viruses in Egypt, characterized by the co-circulation of different subtypes and genotypes in different bird species, in several sectors, and in multiple geographical locations.

Despite the broad scope of the active surveillance implemented within the frame of this study, only a few cases of H5-positive commercial farms (17 out of over 25,000 tested samples) were detected. This might suggest either sufficient biosecurity measures within the commercial poultry farms or limitations in influenza virus detection, as farm owners usually do not report birds showing clinical symptoms. In addition, vaccination is widely used in commercial poultry farms in Egypt, mainly inactivated autogenous vaccines derived from Egyptian H5 strains, in addition to other recombinant H5 vaccines [39,40]. However, updating the vaccine strain to match the circulating clade 2.3.4.4b H5 viruses is not yet widely implemented. Such a poor antigenic match can result in suboptimal immunity in poultry, allowing viral persistence, continued transmission, and the emergence of vaccine escape variants [41] Both H5N1 and H5N8 subtypes were reported throughout the year in all sectors, indicating an enzootic situation for HPAIV H5 in the poultry population in Egypt. Moreover, a relatively higher detection rate in backyard flocks and live bird markets (LBMs) compared to commercial farms, particularly during the winter season (November to March), highlights the climate role for virus maintenance and spread. LBMs in Egypt serve as hubs of viral transmission and reassortment due to the convergence of multiple species under high-density conditions with a lower biosecurity condition [42].

Genetic features of the cleavage site of the HA genes in this study are consistent indicating highly pathogenic avian influenza viruses. Further, receptor-binding site analysis revealed a predominant avian-type (α2,3-linked sialic acid) binding profile according to the HA gene segment analysis. However, the presence of mammalian-adaptive mutations, previously reported in the HA [9] and PA [31] genes of clade 2.3.4.4b highlights the potential mammalian adaptation of those viruses. These findings underscore the importance of continuous genomic surveillance for the early detection of genetic markers that may have implications for cross-species transmission. A limitation of this study is that pathogenicity and the virulence features of the H5 viruses were based on molecular markers without in vivo confirmation. Hence, future experimental studies in chickens and mice are required to better explore these findings. Given the increasing detection of HPAI H5 viruses in various mammal species around the globe, including recent reports in dairy cows, minks, and sea mammals [37] and in wild rats in LBM in Egypt [19], there is a pressing need for vigilance regarding the emergence of antiviral resistance. Phylogenetic analysis aligns with recent European and Eurasian H5 virus genotype classifications, with Egyptian H5N1 viruses clustering with EA-2021-AB-like strains. Genotype H5N1 EA-2020-C has also been reported in previous years in wild birds in Egypt [18]. The dominant H5N8 genotype circulating in Egypt, including viruses for which the whole genome sequence was obtained in this study, is EA-2020-A genotype. The EA-2021-Q genotype was found up to 2019 based on the analysis of publicly available sequence data and not found in the sequences generated in this study.. These findings highlight the continuous virus dissemination and introductions via migratory bird flyways [9]. The detection of at least two variant lineages of Egyptian H5N8 viruses based on amino acid variations further supports separate introductions and independent virus evolution.

Evolutionary analyses revealed that the Egyptian HPAI H5N8 viruses are evolving at a higher nucleotide substitution rate than HPAI H5N1 viruses. This higher rate in H5N8 may be due to its more extensive circulation, broader host range, or stronger immune-driven selection pressure due to the intensive H5N8 vaccination in Egypt. Moreover, MEME and FEL analyses also indicated a significantly higher number of positively selected sites in the HA protein of the Egyptian HPAI H5N8 viruses compared to H5N1, including sites 149 and 152, which were detected by both models. The presence of selective hotspots and accelerated evolution in HPAI H5N8 viruses may signify early signs of antigenic drift, raising concerns about vaccine efficacy [40]. These findings support the necessity of updating antigenic components of HPAI virus vaccines in Egypt to match the circulating strains more closely.

In conclusion, this study provides insights into the epidemiology and evolution of two co-circulating strains of HPAI H5 (H5N1 and H5N8). The continued viral detection in backyards and LBMs, associated with growing genetic diversity and recent reports in mammals, suggests a persistent risk of virus spread and potential spillover to humans. Therefore, in addition to improving biosecurity in different sectors, ongoing studies including, continuous virus monitoring and updated vaccines, remain essential components of effective avian influenza virus control strategies in Egypt.

## Supplementary Material

Supplemental files.docx

## Data Availability

The obtained sequences in this study were submitted to GenBank under the accession number shown in Supplementary Table S2.
