# Supplementary material for: Genomic surveillance and evolution of co-circulating avian influenza H5N1 and H5N8 viruses in Egypt, 2022–2024
Source: Emerg Microbes Infect. 2025 Sep 15;14(1):2562046. doi: 10.1080/22221751.2025.2562046 (PMC12456045; doi:10.1080/22221751.2025.2562046)
Supplement: Supplemental files.docx [file TEMI_A_2562046_SM7734.docx]

Supplementary information for:

**Genomic surveillance and risk mapping of avian influenza A(H5) viruses circulating in Egypt, 2022-2024**

**Supplementary table 1.** Number of examined and positive cases 2022-2024 for A(H5) viruses.

| **Serial** | **Governorates** | **No. of tested cases** | | | **Positive cases** | | |
| --- | --- | --- | --- | --- | --- | --- | --- |
|  |  | **Farms** | **backyards** | **markets** | **Farms** | **backyards** | **markets** |
| 1 | Alexandria | 187 | 2 | 27 | 0 | 0 | 0 |
| 2 | Aswan | 51 | 4 | 90 | 0 | 1 | 3 |
| 3 | Asyut | 313 | 7 | 54 | 0 | 0 | 23 |
| 4 | Beheira | 1674 | 42 | 56 | 0 | 0 | 2 |
| 5 | Beni_Suef | 98 | 1 | 43 | 2 | 0 | 5 |
| 6 | Cairo | 47 | 13 | 0 | 0 | 1 | 0 |
| 7 | Dakahlia | 4138 | 4 | 66 | 1 | 0 | 4 |
| 8 | Damietta | 373 | 6 | 18 | 0 | 0 | 1 |
| 9 | Faiyum | 667 | 20 | 131 | 1 | 1 | 5 |
| 10 | Gharbia | 2991 | 183 | 85 | 3 | 1 | 8 |
| 11 | Giza | 1172 | 5 | 75 | 3 | 0 | 26 |
| 12 | Ismailia | 333 | 4 | 72 | 0 | 0 | 8 |
| 13 | Kafr El Sheikh | 721 | 12 | 28 | 0 | 0 | 1 |
| 14 | Luxor | 74 | 1 | 27 | 0 | 0 | 1 |
| 15 | Matrouh | 14 | 0 | 0 | 0 | 0 | 0 |
| 16 | Minya | 493 | 45 | 55 | 1 | 2 | 20 |
| 17 | Monufia | 2887 | 24 | 69 | 1 | 3 | 13 |
| 18 | New Valley | 1659 | 0 | 0 | 0 | 0 | 0 |
| 19 | North Sinai | 37 | 1 | 14 | 0 | 0 | 0 |
| 20 | Port Said | 68 | 0 | 39 | 0 | 0 | 0 |
| 21 | Qalyubia | 3494 | 10 | 56 | 3 | 1 | 8 |
| 22 | Qena | 124 | 0 | 52 | 0 | 0 | 0 |
| 23 | Red Sea | 200 | 0 | 0 | 0 | 0 | 0 |
| 24 | Sharqia | 2905 | 5 | 107 | 1 | 0 | 0 |
| 25 | Sohag | 199 | 11 | 53 | 1 | 1 | 13 |
| 26 | South Sinai | 38 | 0 | 0 | 0 | 0 | 0 |
| 27 | Suez | 73 | 0 | 33 | 0 | 0 | 4 |
|  | unknown | 27 | 3 | 0 | 0 | 0 | 0 |
| Total |  | 25057 | 403 | 1250 | 17 | 11 | 145 |

**Supplementary table 2**. Epidemiological data and accession number of sequenced viruses.

| **Serial** | **Sample ID** | **Governorate** | **Source** | **Accession no*** |
| --- | --- | --- | --- | --- |
| 1 | A/turkey/Egypt/FAO-S110TH3/2022(H5N1) | Gharbia | LBM | EPI4609667, EPI4611494 |
| 2 | A/turkey/Egypt/FAO-S177/2022(H5N1) | Monufia | LBM | EPI4609668, EPI4611491 |
| 3 | A/turkey/Egypt/FAO-S176/2022(H5N1) | Gharbia | LBM | EPI4609669, EPI4611526 |
| 4 | A/chicken/Egypt/F581/2022 (H5N8) | Giza | FARM | EPI4609677 |
| 5 | A/duck/Egypt/FAO-S1/2022 (H5N8) | Qalyubia | LBM | EPI4609678 |
| 6 | A/turkey/Egypt/FAO-S2-TF3/2022 (H5N8) | Giza | LBM | EPI4609686 |
| 7 | A/turkey/Egypt/FAO-S2-TF2/2022 (H5N8) | Giza | LBM | EPI4609687 |
| 8 | A/duck/Egypt/FAO-S4/2022 (H5N8) | Monufia | LBM | EPI4609679 |
| 9 | A/duck/Egypt/FAO-S5/2022 (H5N8) | Giza | LBM | EPI4609688 |
| 10 | A/duck/Egypt/ FAO-S7/2022 (H5N8) | Asyut | LBM | EPI4609697 |
| 11 | A/duck/Egypt/FAO-S8-DH7/2022 (H5N8) | Minya | LBM | EPI4609681 |
| 12 | A/duck/Egypt/FAO-S8-DH9/2022 (H5N8) | Minya | LBM | EPI4609682 |
| 13 | A/chicken/Egypt/FAO-S9/2022 (H5N8) | Minya | LBM | EPI4609694 |
| 14 | A/chicken /Egypt/FAO-S28/2022 (H5N8) | Monufia | LBM | EPI4609680 |
| 15 | A/ duck/Egypt/ FAO-S29/2022 (H5N8) | Giza | LBM | EPI4609689 |
| 16 | A/chicken/Egypt/FAO-S30/2022 (H5N8) | Giza | LBM | EPI4609690 |
| 17 | A/chicken/Egypt/FAO-S43/2022 (H5N8) | Minya | LBM | EPI4609698 |
| 18 | A/duck/Egypt/ FAO-S53/2022 (H5N8) | Giza | LBM | EPI4609684 |
| 19 | A/chicken/Egypt/FAO-S56/2022 (H5N8) | Gharbia | LBM | EPI4609685, EPI4611504 |
| 20 | A/duck/Egypt/V1506/2022 (H5N8) | Minya | FARM | EPI4609696 |
| 21 | A/chicken/Egypt/F1048/2022 (H5N8) | Giza | FARM | EPI4609695, EPI4609695 |
| 22 | A/duck/Egypt/V1077/2022 (H5N8) | Qalyubia | FARM | EPI4609683 |
| 23 | A/duck/Egypt/FAO-S13/2023 (H5N8) | Asyut | LBM | EPI4609691, EPI4611511 |
| 24 | A/duck/Egypt/FAO-S16/2023 (H5N1) | Qalyubia | LBM | EPI4609672 |
| 25 | A/chicken / Egypt/FAO /Sl40/2023 (H5N8) | BeniSueif | FARM | EPI4609692 |
| 26 | A/turkey/Egypt/FAO-S21/2023(H5N1) | Monufia | LBM | EPI4609675, EPI4611495 |
| 27 | A/chicken/Egypt/F1154/2023 (H5N8) | Giza | FARM | EPI4609693 |
| 28 | A/duck/Egypt/FAO-S111/2023(H5N1) | Giza | LBM | EPI4609673 |
| 29 | A/chicken/Egypt/FAO-S160/2023 (H5N8) | Minya | LBM | EPI4609700 |
| 30 | A/environment/Egypt/FAO-S136/2023(H5N1) | Monufia | LBM | EPI4609671 |
| 31 | A/chicken/Egypt/CV10/2023(H5N1) | Giza | FARM | EPI4609674, EPI4611496 |
| 32 | A/chicken/Egypt/FAO-SI17/2024 (H5N1) | Asyut | FARM | EPI4609676 |
| 33 | A/turkey/Egypt/FAO/S146-TH2/2024(H5N8) | Asyut | FARM | EPI4609701, EPI4611536 |
| 34 | A/geese/Egypt/FAO-S155/2024(H5N1)^#^ | Monufia | LBM | EPI4469864, EPI4469863  EPI4469865, EPI4469861  EPI4469862, EPI4469868  EPI4469866, EPI4469867 |
| 35 | A/chicken/Egypt/S42/2024(H5N1) ^#^ | Monufia | Backyard | EPI4469792, EPI4469797  EPI4469793, EPI4469781  EPI4469796, EPI4469782  EPI4469794, EPI4469843 |
| 36 | A/duck/Egypt/F76/2024(H5N8) ^#^ | Giza | FARM | EPI4469848, EPI4469847  EPI4469849, EPI4469844  EPI4469852, EPI4469845  EPI4469850, EPI4469851 |
| 37 | A/chicken/Egypt/F78/2024(H5N8) ^#^ | Giza | FARM | EPI4469824, EPI4469823  EPI4469825, EPI4469818  EPI4469828, EPI4469821  EPI4469826, EPI4469827 |
| 38 | A/geese/Egypt/S8/2024(H5N1) ^#^ | Monufia | Backyard | EPI4469772, EPI4469771  EPI4469774, EPI4469769  EPI4469779, EPI4469770  EPI4469776, EPI4469778 |

* GISAID Accession number ^#^ Samples where whole genome sequences were generated

**Supplementary Table 3.** Key amino acid analysis within the HA of the Egyptian HPAI H5 viruses sequenced in this study against reference strains

|  | Receptor binding sites | | | | | | | Cleavage site |
| --- | --- | --- | --- | --- | --- | --- | --- | --- |
|  | 107 | 133 | 193 | 216 | 225 | 226 | 228 |  |
| **H5N8** | | | | | | | | |
| A/duck/Chelyabinsk/1207-1/2020 (H5N8) | H | L | N | K | G | Q | G | PLREKRRKRGLF |
| A/chicken/Egypt/F581/2022 (H5N8) | H | L | N | K | G | Q | G | PLREKRRKRGLF |
| A/duck/Egypt/FAO-S1/2022 (H5N8) | H | L | N | K | G | Q | G | PLREKRRKRGLF |
| A/turkey/Egypt/FAO-S2-TF3/2022 (H5N8) | H | L | N | K | G | Q | G | PLREKRRKRGLF |
| A/turkey/Egypt/FAO-S2-TF2/2022 (H5N8) | H | L | N | K | G | Q | G | PLREKRRKRGLF |
| A/duck/Egypt/FAO-S4/2022 (H5N8) | H | L | N | K | G | Q | G | PLREKRRKRGLF |
| A/duck/Egypt/FAO-S5/2022 (H5N8) | H | L | N | K | G | Q | G | PLREKRRKRGLF |
| A/duck/Egypt/ FAO-S7/2022 (H5N8) | H | L | N | K | G | Q | G | PLREKRRKRGLF |
| A/duck/Egypt/FAO-S8-DH7/2022 (H5N8) | H | L | N | K | G | Q | G | PLREKRRKRGLF |
| A/duck/Egypt/FAO-S8-DH9/2022 (H5N8) | H | L | N | K | G | Q | G | PLREKRRKRGLF |
| A/chicken/Egypt/FAO-S9/2022 (H5N8) | H | L | N | K | G | Q | G | PLREKRRKRGLF |
| A/chicken /Egypt/FAO-S28/2022 (H5N8) | H | L | N | K | G | Q | G | PLREKRRKRGLF |
| A/ duck/Egypt/ FAO-S29/2022 (H5N8) | H | L | N | K | G | Q | G | PLREKRRKRGLF |
| A/chicken/Egypt/FAO-S30/2022 (H5N8) | H | L | N | K | G | Q | G | PLREKRRKRGLF |
| A/chicken/Egypt/FAO-S43/2022 (H5N8) | H | L | N | K | G | Q | G | PLREKRRKRGLF |
| A/duck/Egypt/ FAO-S53/2022 (H5N8) | H | L | N | K | G | Q | G | PLREKRRKRGLF |
| A/chicken/Egypt/FAO-S56/2022 (H5N8) | H | L | N | K | G | Q | G | PLREKRRKRGLF |
| A/duck/Egypt/V1506/2022 (H5N8) | H | L | N | K | G | Q | G | PLREKRRKRGLF |
| A/chicken/Egypt/F1048/2022 (H5N8) | H | L | N | K | G | Q | G | PLREKRRKRGLF |
| A/duck/Egypt/V1077/2022 (H5N8) | H | L | N | K | G | Q | G | PLREKRRKRGLF |
| A/duck/Egypt/FAO-S13/2023 (H5N8) | H | L | N | K | G | Q | G | PLREKRRKRGLF |
| A/chicken / Egypt/FAO /Sl40/2023 (H5N8) | H | L | N | K | G | Q | G | PLREKRRKRGLF |
| A/chicken/ Egypt/F1154/2023 (H5N8) | H | L | N | K | G | Q | G | PLREKRRKRGLF |
| A/chicken/Egypt/FAO-S160/2023 (H5N8) | H | L | N | K | G | Q | G | PLREKRRKRGLF |
| A/turkey/Egypt/FAO/S146-TH2/2024 (H5N8) | H | L | N | **R** | G | Q | G | PLREKRRKRGLF |
| **H5N1** | | | | | | | | |
| A/duck/Saratov/29-02V/2021 (H5N1) | H | L | N | K | G | Q | G | PLREKRRKRGLF |
| A/turkey/Egypt/FAO-S110TH3/2022 (H5N1) | H | L | **K**^1^ | K | G | Q | G | PLREKRRKRGLF |
| A/turkey/Egypt/FAO-S177/2022 (H5N1)) | H | L | **K** | K | G | Q | G | PLREKRRKRGLF |
| A/turkey/Egypt/FAO-S176/2022 (H5N1) | H | L | **K** | K | G | Q | G | PLREKRRKRGLF |
| A/turkey/Egypt/FAO-S21/2023 (H5N1)) | H | L | **K** | K | G | Q | G | PLREKRRKRGLF |
| A/duck/Egypt/FAO-S111/2023 (H5N1) | H | L | **K** | K | G | Q | G | PLREKRRKRGLF |
| A/environment/Egypt/FAO-S136/2023 (H5N1) | H | L | **K** | K | G | Q | G | PLREKRRKRGLF |
| A/chicken/Egypt/CV10/2023(H5N1) | H | L | **K** | K | G | Q | G | PLREKRRKRGLF |
| A/duck/Egypt/FAO-S16/2023 (H5N1) | H | L | **K** | K | G | Q | G | PLREKRRKRGLF |
| A/chicken/Egypt/FAO-SI17/2024 (H5N1) | H | L | **K** | K | G | Q | G | PLREKRRKRGLF |

^1^Bold means different compared to the reference strain

**Supplementary Table 4**. Genin genotyping of the Egyptian HPAI H5N1 and H5N8 viruses generated in this study

| **Sample ID** | **Subtype** | **Clade** | **Genotype** | **PB2** | **PB1** | **PA** | **NP** | **NA** | **MP** | **NS** |
| --- | --- | --- | --- | --- | --- | --- | --- | --- | --- | --- |
| A/chicken/Egypt/F78/2024 | H5N8 | 2.3.4.4b | EA-2020-A | 20* | 20 | 20 | 20 | 20 | 20 | 20 |
| A/duck/Egypt/F76/2024 | H5N8 | 2.3.4.4b | EA-2020-A | 20 | 20 | 20 | 20 | 20 | 20 | 20 |
| A/geese/Egypt/S8/2024 | H5N1 | 2.3.4.4b | EA-2021-AB | 31 | 1 | 3 | 38 | 1 | 20 | 1 |
| A/geese/Egypt/FAO-S155/2024 | H5N1 | 2.3.4.4b | EA-2021-AB | 31 | 1 | 3 | 38 | 1 | 20 | 1 |
| A/chicken/Egypt/S42/2024 | H5N1 | 2.3.4.4b | EA-2021-AB | 31 | 1 | 3 | 38 | 1 | 20 | 1 |

*Number of gene version according to [[9](#_ENREF_9)].

**Supplementary Table 4**. Genin genotyping of the Egyptian HPAI H5N8 viruses publicly available at the GISAID platform.

| **Sample Name** | **Genotype** | **PB2** | **PB1** | **PA** | **NP** | **NA** | **MP** | **NS** |
| --- | --- | --- | --- | --- | --- | --- | --- | --- |
| EPI_ISL_14770153_A/chicken/Egypt/F13829B/2017 | [unassigned] | 20 | 29 | ? | 26 | 20 | 20 | 20 |
| EPI_ISL_4069678_A/chicken/Egypt/Q16710C/2019 | [unassigned] | 20 | 20 | 20 | 38 | 20 | 20 | 20 |
| EPI_ISL_9594290_A/chicken/Egypt/A19670/2021 | [unassigned] | ? | 20 | 20 | 20 | 20 | 20 | 20 |
| EPI_ISL_17508648_A/common teal/Egypt/MB-F-1197OP/2017 | [unassigned] | 20 | 29 | ? | 26 | 20 | 20 | 20 |
| EPI_ISL_9594294_A/chicken/Egypt/N15173A/2018 | EA-2021-Q | 20 | 20 | 20 | 26 | 20 | 20 | 20 |
| EPI_ISL_9594292_A/chicken/Egypt/N15174C/2018 | EA-2021-Q | 20 | 20 | 20 | 26 | 20 | 20 | 20 |
| EPI_ISL_4070936_A/chicken/Egypt/F17229B/2019 | [unassigned] | 20 | 53 | ? | ? | ? | 20 | 20 |
| EPI_ISL_9594293_A/chicken/Egypt/N15175C/2018 | EA-2021-Q | 20 | 20 | 20 | 26 | 20 | 20 | 20 |
| EPI_ISL_9594298_A/chicken/Egypt/N15176A/2018 | EA-2021-Q | 20 | 20 | 20 | 26 | 20 | 20 | 20 |
| EPI_ISL_9594299_A/chicken/Egypt/N15168C/2018 | EA-2021-Q | 20 | 20 | 20 | 26 | 20 | 20 | 20 |
| EPI_ISL_4070932_A/duck/Egypt/N16721/2019 | [unassigned] | 20 | ? | 20 | 26 | 20 | 20 | 20 |
| EPI_ISL_4069652_A/chicken/Egypt/Q16711B/2019 | [unassigned] | 20 | 20 | 20 | 38 | 20 | 20 | 20 |
| EPI_ISL_9594297_A/chicken/Egypt/N15178B/2018 | EA-2021-Q | 20 | 20 | 20 | 26 | 20 | 20 | 20 |
| EPI_ISL_9594302_A/chicken/Egypt/F15100/2018 | EA-2021-Q | 20 | 20 | 20 | 26 | 20 | 20 | 20 |
| EPI_ISL_17508647_A/common teal/Egypt/MB-D-2060OP/2019 | EA-2021-Q | 20 | 20 | 20 | 26 | 20 | 20 | 20 |
| EPI_ISL_4069650_A/chicken/Egypt/N16732/2019 | EA-2020-A | 20 | 20 | 20 | 20 | 20 | 20 | 20 |
| EPI_ISL_4069651_A/duck/Egypt/N16719/2019 | EA-2021-Q | 20 | 20 | 20 | 26 | 20 | 20 | 20 |
| EPI_ISL_9594303_A/chicken/Egypt/N15173C/2018 | EA-2021-Q | 20 | 20 | 20 | 26 | 20 | 20 | 20 |
| EPI_ISL_9594301_A/chicken/Egypt/N15174A/2018 | EA-2021-Q | 20 | 20 | 20 | 26 | 20 | 20 | 20 |
| EPI_ISL_505399_A/chicken/Al-Sharqia/1822FM/2018 | [unassigned] | 20 | 20 | 20 | 50 | 20 | 20 | 20 |
| EPI_ISL_387135_A/duck/Egypt/N13736E/2017 | [unassigned] | 20 | 29 | ? | 26 | 20 | 20 | 20 |
| EPI_ISL_4069758_A/duck/Egypt/N16717/2019 | EA-2021-Q | 20 | 20 | 20 | 26 | 20 | 20 | 20 |
| EPI_ISL_387141_A/northern shoveler/Egypt/813C/2016 | [unassigned] | ? | 20 | ? | ? | 20 | 20 | 20 |
| EPI_ISL_4069759_A/chicken/Egypt/Q16712A/2019 | [unassigned] | 20 | 20 | 20 | 38 | 20 | 20 | 20 |
| EPI_ISL_387140_A/teal/Egypt/1202C/2017 | [unassigned] | 20 | 29 | ? | 26 | 20 | 20 | 20 |
| EPI_ISL_4069756_A/chicken/Egypt/Q16711C/2019 | [unassigned] | 20 | 20 | 20 | 38 | 20 | 20 | 20 |
| EPI_ISL_505413_A/chicken/Albehra/18319F/2018 | [unassigned] | 20 | 20 | 20 | 50 | 20 | 20 | 20 |
| EPI_ISL_505411_A/Turkey/Beni Sueif/18296F/2018 | [unassigned] | 20 | 20 | 20 | 50 | 20 | 20 | 20 |
| EPI_ISL_387137_A/chicken/Egypt/Q13804A/2017 | [unassigned] | 20 | 29 | ? | 26 | 20 | 20 | 20 |
| EPI_ISL_387136_A/teal/Egypt/1198C/2017 | [unassigned] | 20 | 29 | ? | 26 | 20 | 20 | 20 |
| EPI_ISL_4069752_A/chicken/Egypt/N16730/2019 | EA-2020-A | 20 | 20 | 20 | 20 | 20 | 20 | 20 |
| EPI_ISL_387139_A/duck/Egypt/F13666A/2017 | [unassigned] | 20 | 29 | ? | 26 | 20 | 20 | 20 |
| EPI_ISL_387138_A/teal/Egypt /823C/2016 | [unassigned] | ? | 20 | ? | ? | 20 | 20 | 20 |
| EPI_ISL_4069750_A/duck/Egypt/N16720/2019 | EA-2021-Q | 20 | 20 | 20 | 26 | 20 | 20 | 20 |
| EPI_ISL_9594306_A/chicken/Egypt/S18182C/2020 | [unassigned] | ? | 20 | ? | 37 | 20 | 20 | 20 |
| EPI_ISL_9594307_A/chicken/Egypt/N15178D/2018 | EA-2021-Q | 20 | 20 | 20 | 26 | 20 | 20 | 20 |
| EPI_ISL_9594305_A/chicken/Egypt/N15173B/2018 | EA-2021-Q | 20 | 20 | 20 | 26 | 20 | 20 | 20 |
| EPI_ISL_9594310_A/chicken/Egypt/N15176B/2018 | EA-2021-Q | 20 | 20 | 20 | 26 | 20 | 20 | 20 |
| EPI_ISL_9594311_A/chicken/Egypt/N15169A/2018 | EA-2021-Q | 20 | 20 | 20 | 26 | 20 | 20 | 20 |
| EPI_ISL_9594308_A/chicken/Egypt/N15178C/2018 | EA-2021-Q | 20 | 20 | 20 | 26 | 20 | 20 | 20 |
| EPI_ISL_4069736_A/duck/Egypt/Q16716A/2019 | EA-2021-Q | 20 | 20 | 20 | 26 | 20 | 20 | 20 |
| EPI_ISL_9594309_A/chicken/Egypt/N15178A/2018 | EA-2021-Q | 20 | 20 | 20 | 26 | 20 | 20 | 20 |
| EPI_ISL_14770049_A/duck/Egypt/N14200A/2017 | [unassigned] | 20 | 29 | ? | 26 | 20 | 20 | 20 |
| EPI_ISL_14770051_A/chicken/Egypt/F14110D/2017 | [unassigned] | 20 | 20 | 20 | 38 | 20 | 20 | 20 |
| EPI_ISL_4069710_A/chicken/Egypt/Q16711A/2019 | [unassigned] | 20 | 20 | 20 | 38 | 20 | 20 | 20 |
| EPI_ISL_14937233_A/Turkey/Egypt/Giza/2021 | EA-2020-A | 20 | 20 | 20 | 20 | 20 | 20 | 20 |
| EPI_ISL_267135_A/green-winged teal/Egypt/871/2016 | [unassigned] | ? | 20 | ? | 37 | 20 | 20 | 20 |
| EPI_ISL_16209015_A/chicken/Egypt/FAOS18/2020 | [unassigned] | 20 | 20 | 20 | 50 | 20 | 20 | 28 |
| EPI_ISL_4070847_A/chicken/Egypt/F17230B/2019 | EA-2020-A | 20 | 20 | 20 | 20 | 20 | 20 | 20 |
| EPI_ISL_18847132_A/common pochard/Egypt/SJCEIRR-DT19799C/2021 | EA-2020-A | 20 | 20 | 20 | 20 | 20 | 20 | 20 |
| EPI_ISL_18847131_A/duck/Egypt/SJCEIRR-BA19903OP/2021 | EA-2020-A | 20 | 20 | 20 | 20 | 20 | 20 | 20 |
| EPI_ISL_267136_A/green-winged teal/Egypt/877/2016 | [unassigned] | ? | 20 | ? | 37 | 20 | 20 | 20 |
| EPI_ISL_18847130_A/duck/Egypt/SJCEIRR-BA20036OP/2022 | EA-2020-A | 20 | 20 | 20 | 20 | 20 | 20 | 20 |
| EPI_ISL_14770020_A/chicken/Egypt/Q16684C/2019 | EA-2021-Q | 20 | 20 | 20 | 26 | 20 | 20 | 20 |
| EPI_ISL_14770033_A/chicken/Egypt/N13731D/2017 | [unassigned] | 20 | 29 | ? | 26 | 20 | 20 | 20 |
| EPI_ISL_885148_A/Chicken/Egypt/F1366A/2017 | [unassigned] | 20 | 29 | ? | 26 | 20 | 20 | 20 |
| EPI_ISL_14770035_A/duck/Egypt/N13736A/2017 | [unassigned] | 20 | 29 | ? | 26 | 20 | 20 | 20 |
| EPI_ISL_14770037_A/chicken/Egypt/Q13845A/2017 | [unassigned] | 20 | 29 | ? | 26 | 20 | 20 | 20 |
| EPI_ISL_14770036_A/chicken/Egypt/H13794D/2017 | [unassigned] | 20 | 29 | ? | 26 | 20 | 20 | ? |
| EPI_ISL_14770039_A/chicken/Egypt/A13773E/2017 | [unassigned] | 20 | 29 | ? | 26 | 20 | 20 | ? |
| EPI_ISL_17508498_A/common teal/Egypt/MB-D-2066OP/2019 | EA-2021-Q | 20 | 20 | 20 | 26 | 20 | 20 | 20 |
| EPI_ISL_14770043_A/chicken/Egypt/Q13936C/2017 | [unassigned] | 20 | 20 | ? | 26 | 20 | 20 | ? |
| EPI_ISL_17508496_A/common teal/Egypt/MB-D-2065OP/2019 | EA-2021-Q | 20 | 20 | 20 | 26 | 20 | 20 | 20 |
| EPI_ISL_14770042_A/duck/Egypt/N14205C/2017 | EA-2021-Q | 20 | 20 | 20 | 26 | 20 | 20 | 20 |
| EPI_ISL_400027_A/Turkey/Egypt/AI20285/2019 | EA-2021-Q | 20 | 20 | 20 | 26 | 20 | 20 | 20 |
| EPI_ISL_14770047_A/chicken/Egypt/N15172D/2018 | EA-2021-Q | 20 | 20 | 20 | 26 | 20 | 20 | 20 |
| EPI_ISL_17508500_A/common teal/Egypt/MB-D-2062OP/2019 | EA-2021-Q | 20 | 20 | 20 | 26 | 20 | 20 | 20 |
| EPI_ISL_14770046_A/chicken/Egypt/N13720E/2017 | [unassigned] | 20 | 29 | ? | 26 | 20 | 20 | 20 |
| EPI_ISL_388773_A/little grebe/Egypt/1056OP/2016 | [unassigned] | ? | 20 | ? | 37 | 20 | 20 | 20 |
| EPI_ISL_17508515_A/common teal/Egypt/MB-F-1201C/2017 | [unassigned] | 20 | 29 | ? | 26 | 20 | 20 | 20 |
| EPI_ISL_4069778_A/duck/Egypt/A16793/2019 | EA-2020-A | 20 | 20 | 20 | 20 | 20 | 20 | 20 |
| EPI_ISL_17508519_A/common teal/Egypt/MB-F-1198OP/2017 | [unassigned] | 20 | 29 | ? | 26 | 20 | 20 | 20 |
| EPI_ISL_17508518_A/common teal/Egypt/MB-F-1202OP/2017 | [unassigned] | 20 | 29 | ? | 26 | 20 | 20 | 20 |
| EPI_ISL_400043_A/Chicken/Egypt/AI20286/2019 | EA-2021-Q | 20 | 20 | 20 | 26 | 20 | 20 | 20 |
| EPI_ISL_17508517_A/common teal/Egypt/MB-F-1196C/2017 | [unassigned] | 20 | 29 | ? | 26 | 20 | 20 | 20 |
| EPI_ISL_285620_A/chicken/Egypt/Buheira-12/2017 | [unassigned] | 20 | 29 | 20 | 26 | 20 | 20 | 20 |
| EPI_ISL_285621_A/chicken/Egypt/Gharbiya-15/2017 | EA-2021-Q | 20 | 20 | 20 | 26 | 20 | 20 | 20 |
| EPI_ISL_285622_A/duck/Egypt/Buheira-21/2017 | [unassigned] | 20 | 29 | ? | 26 | 20 | 20 | 20 |
| EPI_ISL_17509823_A/common teal/Egypt/MB-F-1205C/2017 | [unassigned] | 20 | 29 | ? | 26 | 20 | 20 | 20 |
| EPI_ISL_17509822_A/common teal/Egypt/MB-F-1200OP/2017 | [unassigned] | 20 | 29 | ? | 26 | 20 | 20 | 20 |
| EPI_ISL_9572645_A/chicken/Egypt/N13731C/2017 | [unassigned] | 20 | 29 | ? | 26 | 20 | 20 | 20 |
| EPI_ISL_285619_A/chicken/Egypt/Kafr-Elshiekh-18/2017 | [unassigned] | 20 | 20 | ? | 26 | 20 | 20 | 20 |
| EPI_ISL_4069765_A/chicken/Egypt/Q16807E/2019 | EA-2021-Q | 20 | 20 | 20 | 26 | 20 | 20 | 20 |
| EPI_ISL_4069762_A/duck/Egypt/N16722/2019 | EA-2021-Q | 20 | 20 | 20 | 26 | 20 | 20 | 20 |
| EPI_ISL_17509835_A/common teal/Egypt/MB-F-1204OP/2017 | [unassigned] | 20 | 29 | ? | 26 | 20 | 20 | 20 |
| EPI_ISL_17509834_A/common teal/Egypt/MB-F-1204C/2017 | [unassigned] | 20 | 29 | ? | 26 | 20 | 20 | 20 |
| EPI_ISL_17509837_A/common teal/Egypt/MB-F-1199OP/2017 | [unassigned] | 20 | 29 | ? | 26 | 20 | 20 | 20 |
| EPI_ISL_4137976_A/chicken/Egypt/AF12/2020 | [unassigned] | 20 | 53 | 20 | 20 | 20 | 20 | 20 |
| EPI_ISL_17509836_A/common teal/Egypt/MB-D-2060C/2019 | EA-2021-Q | 20 | 20 | 20 | 26 | 20 | 20 | 20 |
| EPI_ISL_17509826_A/common teal/Egypt/MB-P-871C/2016 | [unassigned] | ? | 20 | ? | 37 | 20 | 20 | 20 |
| EPI_ISL_344527_A/Duck/Egypt/AR518/2017 | [unassigned] | 20 | 29 | ? | 26 | 20 | 20 | 20 |
| EPI_ISL_17509824_A/common teal/Egypt/MB-D-2063OP/2019 | EA-2021-Q | 20 | 20 | 20 | 26 | 20 | 20 | 20 |
| EPI_ISL_17509829_A/common teal/Egypt/MB-D-829OP/2016 | [unassigned] | ? | 20 | ? | ? | 20 | 20 | 20 |
| EPI_ISL_17509828_A/common teal/Egypt/MB-F-1203OP/2017 | [unassigned] | 20 | 29 | ? | 26 | 20 | 20 | 20 |
| EPI_ISL_4070379_A/chicken/Egypt/Q16710B/2019 | [unassigned] | 20 | 20 | 20 | 38 | 20 | 20 | 20 |
| EPI_ISL_344541_A/Chicken/Egypt/AR553/2018 | EA-2021-Q | 20 | 20 | 20 | 26 | 20 | 20 | 20 |
| EPI_ISL_17509840_A/common teal/Egypt/MB-F-1199C/2017 | [unassigned] | 20 | 29 | ? | 26 | 20 | 20 | 20 |
| EPI_ISL_344539_A/Turkey/Egypt/AR550/2018 | [unassigned] | 20 | ? | 20 | 26 | 20 | 20 | 20 |
| EPI_ISL_268513_A/duck/Egypt/F446/2017 | [unassigned] | 20 | 20 | 20 | 26 | ? | 20 | 20 |
| EPI_ISL_344546_A/Duck/Egypt/AR560/2018 | [unassigned] | 20 | ? | ? | 26 | 20 | 20 | 20 |
| EPI_ISL_268021_A/duck/Egypt/SS19/2017 | [unassigned] | 20 | 29 | ? | 26 | ? | 20 | 28 |

**Supplementary Table 6**. Genin genotyping of the Egyptian HPAI H5N1 viruses publicly available at the GISAID platform.

| Sample Name | Genotype | PB2 | PB1 | PA | NP | NA | MP | NS |
| --- | --- | --- | --- | --- | --- | --- | --- | --- |
| EPI_ISL_18847167_A/garganey/Egypt/SJCEIRR-RA20851OP/2022 | [unassigned] | 14 | 1 | 14 | 11 | 1 | 20 | 27 |
| EPI_ISL_18846973_A/duck/Egypt/SJCEIRR-PS21060C/2023 | EA-2021-AB | 31 | 1 | 3 | 38 | 1 | 20 | 1 |
| EPI_ISL_18846972_A/duck/Egypt/SJCEIRR-PS21060OP/2023 | EA-2021-AB | 31 | 1 | 3 | 38 | 1 | 20 | 1 |
| EPI_ISL_18846971_A/duck/Egypt/SJCEIRR-PS21061C/2023 | EA-2021-AB | 31 | 1 | 3 | 38 | 1 | 20 | 1 |
| EPI_ISL_18846970_A/duck/Egypt/SJCEIRR-PS21061OP/2023 | EA-2021-AB | 31 | 1 | 3 | 38 | 1 | 20 | 1 |
| EPI_ISL_18847129_A/duck/Egypt/SJCEIRR-BA20360C/2022 | EA-2021-AB | 31 | 1 | 3 | 38 | 1 | 20 | 1 |
| EPI_ISL_18846969_A/duck/Egypt/SJCEIRR-RA20838OP/2022 | EA-2021-AB | 31 | 1 | 3 | 38 | 1 | 20 | 1 |
| EPI_ISL_18846968_A/duck/Egypt/SJCEIRR-RA20839OP/2022 | EA-2021-AB | 31 | 1 | 3 | 38 | 1 | 20 | 1 |
| EPI_ISL_17529371_A/duck/Egypt/BA20361OP/2022 | EA-2021-AB | 31 | 1 | 3 | 38 | 1 | 20 | 1 |
| EPI_ISL_17529370_A/duck/Egypt/BA20360OP/2022 | EA-2021-AB | 31 | 1 | 3 | 38 | 1 | 20 | 1 |
| EPI_ISL_17529369_A/duck/Egypt/BA20360C/2022 | EA-2021-AB | 31 | 1 | 3 | 38 | 1 | 20 | 1 |
| EPI_ISL_17529372_A/duck/Egypt/RA19853OP/2021 | EA-2021-AB | 31 | 1 | 3 | 38 | 1 | 20 | 1 |
| EPI_ISL_15614803_A/ibis/Egypt/RLQP-229S/2022 | EA-2020-C | 1 | 1 | 1 | 1 | 1 | 20 | 1 |
| EPI_ISL_18847168_A/garganey/Egypt/SJCEIRR-DT20899OP/2022 | EA-2021-AB | 31 | 1 | 3 | 38 | 1 | 20 | 1 |

**Supplementary Table 7.** Amino acid sites under positive selection pressure of the HA of H5N1 and H5N8 in Egypt estimated using different analytical methods.

| Gene | a.a residue | MEME | | FEL | |
| --- | --- | --- | --- | --- | --- |
|  |  | ‎(P-value <= 0.1)‎ | Number of Branches under Episodic Selection | ‎(P-value <= 0.1)‎ | Likelihood Ratio Test (LRT) |
| H5N1 | 84 | 0.021 | 1 | - | - |
| H5N8 | 47 | 0.0011 | ‎1 | - | - |
|  | 94 | 0.0056 | ‎1 | - | - |
|  | 95 | 0.0000 | ‎4 | - | - |
|  | 133 | 0.0004 | ‎5 | - | - |
|  | **137** | **0.0478** | **4** | **0.0840** | **2.986** |
|  | 163 | 0.0998 | 4 | 0.0807 | 3.051 |
|  | 314 | 0.0411 | ‎2 | - | - |
|  | 327 | 0.0409 | ‎2 | - | - |
|  | 339 | 0.0218 | ‎6 | - | - |

Amino Acids (a.a) detect in both methods are shown in bold.

**Supplementary figure 1.** Phylogenetic relationships of the internal gene segments of Egyptian and representative H5N1 and H5N8 viruses. Viruses generated in this study is shown by black dots. Representative reference strains from different genotypes are shown in black colour. Trees were generated, after the selection of the best-fitted model, by employing maximum likelihood methodology based on Akaike criterion using IQ-tree software version 1.1.3 [[27](#_ENREF_27)].

**
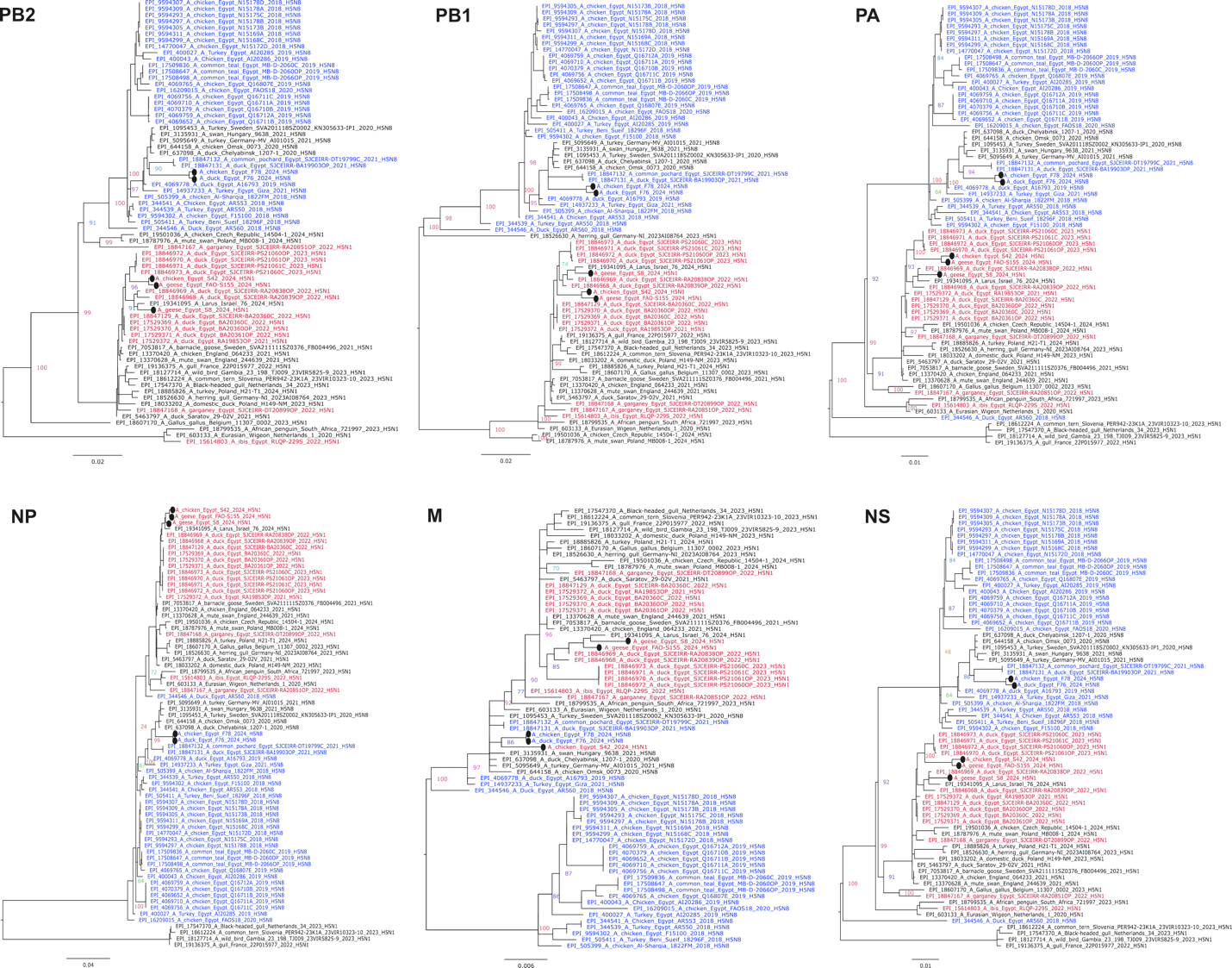
**
